# Pathomolecular characterization of recently isolated duck Astrovirus from domestic ducklings in Egypt

**DOI:** 10.1038/s41598-026-50596-x

**Published:** 2026-05-22

**Authors:** Eman M.S. EL-Nagar, Maha A.N. Gamal, Mohamed A. El-Saied, Mohamed R. Mousa, Karim Selim, Heba M. Salem

**Affiliations:** 1 Genetic Engineering Research Department, Veterinary Serum and Vaccine Research Institute (VSVRI), Agricultural Research Center (ARC), Cairo, 11381 Egypt; 2 Biotechnology Department, Central Laboratory for Evaluation of Veterinary Biologics (CLEVB), Agricultural Research Center (ARC), Cairo, 11381 Egypt; 3 Department of Pathology, Faculty of Veterinary Medicine, Cairo University, Giza, 12211 Egypt; 4 Referance Laboratory for Quality Control on poultry production, Agriculture Research Center (ARC), Giza, 12618 Egypt; 5 Department of Poultry Diseases, Faculty of Veterinary Medicine, Cairo University, Giza, 12211 Egypt; 6 Department of Diseases of Birds, Rabbits, Fish & their Care & Wildlife, School of Veterinary Medicine, Badr University in Cairo (BUC), Badr City, Egypt

**Keywords:** Astro virus, Ducks, Hepatitis, Histopathology, Opisthotonos, Sequencing, Molecular biology, Diseases, Pathogenesis

## Abstract

Astrovirus infection significantly impacts poultry production due to its fatal effect on young birds and their ability to be transmitted by horizontal and vertical routes. In the current study, eight Egyptian duckling farms from different breeds (Moulard, Muscovy, and Pekin) with a history of sudden death accompanied by neurological signs with marked hepatitis lesions at Al-Qalyubia, Al-Sharqia, and Assuit governorates from 2022 to 2023 have been investigated. All the examined freshly dead ducklings have a history of vaccination with the live attenuated duck hepatitis vaccine. During the laboratory investigations, DAstV was successfully isolated on the chorioallantoic membrane (CAM) of specific pathogen-free (SPF) embryonated chicken eggs (ECE) and designated as DAstV- VSVRI-CLEVB-2022 and showed the characteristic star-shaped six-point end of astrovirus when examined under the electron microscope. Molecular identification showed positive PCR amplification targeting the 434 bp of the ORF1b encoding non-structural proteins and is highly conserved of the G4260 Avastrovirus 2, sequence analysis showed that the isolated strain showed 100% similarity with duck astrovirus strain HBGT-China-2014 (KX290465.1). Pathogenicity testing of the isolated astrovirus in susceptible one-day-old ducklings, showed typical death signs of astrovirus at 31, 38, and 50 h post-infection (PI) with paint brush hemorrhagic liver surface. The above-mentioned results showed that DAstV could be one of the causative agents of acute duck hepatitis in Egypt and further investigations are recommended to prepare a local autogenous vaccine from the isolated strain with the application of strict biosecurity measures and evaluate its efficacy against the field challenge to control such a problem.

## Introduction

 The World Organization for Animal Health (WOAH) categorized duck viral hepatitis (DVH) as an acute, highly contagious, and notifiable viral infection attacking ducklings, causing mainly nervous manifestations accompanied by high morbidity and mortality rates that reveal significant financial losses in duck production across the globe^1,2^. Since 1950, both classical and new duck Hepatotropic viruses have been detected, causing the disease historically known as duck hepatitis (DH) or duck viral Hepatitis (DVH)^3^. Historically the nomenclature of DVH was not a problem but recently, there is a complexity and irrationality of the disease name due to the presence of 14 prototypes of viruses associated in this disease, including 6 prototypes in the *Picornaviridae* family, 3 prototypes in the *Astroviridae* family, 2 prototypes in the *Flaviviridae* family, and 1 prototype each in the *Hepadnaviridae* family, the *Kolmioviridae* family, and the *Hepeviridae* family. In total, they belong to 10 genera among 6 viral families^4^.

DVH was historically subdivided into types I, II, and III^5^. Based on genetic evidence and International Committee on Taxonomy of Viruses **(**ICTV**)** classification^6^ DVH type I is caused by one of at least three genotypes (DHAV-1, DHAV-2 and DHAV-3) of duck hepatitis A virus (DHAV) of the genus *Avihepatovirus* in the *Picornaviridae* family. The most widespread and virulent is DHAV type 1 (DHAV-1)^7–9^.

DVH type II and DVH type III caused by duck astrovirus-1 (DAstV-1), and duck astrovirus-2 (DAstV-2) are considered an acute, fatal infection of ducklings producing clinical and pathological signs like DHAV in the family *Astroviridae* in order^10,11^. The genomic sequence of type I DHVs is greatly different from type II and III DHVs with high evolutionary distance^12^.

Astroviruses (AstVs) are members of the *Astroviridae* family, they are infectious, non-enveloped, positive-sense, single-stranded RNA viruses that infect a diverse host range. The *Astroviridae* family is classified into two genera based on their host range: mamastrovirus and avastrovirus. According to the ICTV, the two genera contain 22 species, namely Mamastroviruses 1–19 and Avastroviruses 1–3^13^. Avastrovirus 1 (turkey astrovirus 1), Avastrovirus 2 (avian nephritis virus 1 and avian nephritis virus 2), and Avastrovirus 3 (turkey astrovirus 2 and duck astrovirus 1) are all members of the Avastroviruses genus. These avian viruses have been related to a variety of avian disorders, including hepatitis^14^, enteritis^15^, runting-stunting syndrome^16^, white chicken syndrome^17^, and gout^18^. Furthermore, several other newly discovered astrovirus genotypes have not been classified into any avastrovirus species; however, they have been suggested to the ICTV as unassigned species^19^.

Duck astroviruses have five genotypes: duck astrovirus 1 (DAstV-1), duck astrovirus 2 (DAstV-2), duck astrovirus 3 (DAstV-3), and duck astrovirus 4 (DAstV-4) and these four duck astroviruses differ genetically from one another^20^. Duck hepatitis 2 (DHV-2) and duck hepatitis 3 (DHV-3) are caused by DAstV-1 and DAstV-2, respectively^20^. In 2013, DAstV-3 was discovered in newly hatched, healthy Pekin ducklings^21^. DAstV-4 was isolated from Pekin ducks in Guangdong province’s live-bird markets^22^. However, DAstV-3 and DAstV-4 have not been linked to any diseases, the fifth genotype duck astrovirus (DAstV-5) was discovered by Zhang et al.^19^. during their work, they discovered a novel duck astrovirus in ducklings suffering from gout disease, and till their discovery, no case of duck astrovirus-induced gout has been reported.

DAstV, like DHV, is characterized clinically by the rapid onset of the nervous manifestation “opisthotonos” accompanied by high mortalities, and the postmortem lesions are distinguished mainly by hepatomegaly with ecchymosis and petechial hemorrhages, brain congestion with haemorrhages, splenomegaly with mottled necrosis, and focal pancreatic necrosis^23^. Differentiation between DAstV and DHAV using by polymerase chain reaction and by inoculation on embryonating chicken eggs where DASTV can grow on Chorio allantoic membrane (CAM) of 11 day old ECE and produce necrotic lesions while DHAV grow on allantoic sac of ECE and can’t develop any lesion on CAM**)**^24^. Despite the regular vaccination of both duck breeder flocks at 8 weeks of age and 2 weeks before the laying period to provide sufficient maternal derived antibodies and ducklings during the first week of life if they obtained from un-vaccinated breeders against DVH, it continued to attack ducklings throughout six weeks of age^25,26^. Researchers attempted to employ an emergency DVH-serotype-1 vaccination for duckling during the outbreak and discovered that it was protective, with no mortalities of experimentally infected ducklings with DVH following emergency vaccination until the end of the trial (40 days)^27^. In contrast to the artificially infected and not emergently vaccinated ducklings, mortality reached up to 40% accompanied by severe hepatic lesions^27^. In Egypt, DHAV-1 and DHAV-3 are the most reported serotypes, causing huge economic losses^28,29^. In the present study, astrovirus infection was recorded in Egyptian ducklings suffering from acute hepatic lesions and nervous manifestations, the isolated virus was subjected to molecular and sequence analysis, also histopathological and pathological changes due to the field infection, and experimental infection in susceptible ducklings were studied.

## Materials and methods

### Ethical Statement

Birds were reared and handled in accordance with the guidelines and regulations the Institutional Animal Care and Use Committee (IACUC), under ethical approval number Vet CU “03162023704”. All methods were performed in accordance with relevant guidelines and regulations. Bird handling was performed by experienced veterinarians, and all experimental procedures adhered to ARRIVE guideline 2.0 for the care and use of laboratory animals.

### Samples collection

The samples used in this study were submitted directly by farm owners to our laboratory for routine diagnostic investigation of deceased ducklings. Owners provided consent for the use of these samples in diagnostic procedures and related research activities to reach an accurate diagnosis. Tissue samples, including liver, spleen, brain, and pancreas, have been collected from freshly dead duckling due to natural field infection (five ducklings/farm) with a history of sudden death with neurological signs from different Egyptian governorates (Al-Qalyubia, Al-Sharqia, and Assuit) during the period from 2022 to 2023 (Table 1). All samples were obtained from different farms of different breeding facilities. Ducklings were suffering from nervous manifestations and showed severe hepatic lesions during postmortem (PM) examination. All dead ducklings were vaccinated with live attenuated duck hepatitis vaccine at the first week of age. Liver samples were tested molecularly to exclude the other virulent waterfowl associated viruses (H5N1 virus infection)^24^.

**Table 1:** The history of the investigated suspected duck farms.

Samples code	Date of collected samples	Location	Breed	Age	Farm capacity	Mortalities recorded during the last days during farm visit
H1	2022	Qalyubia	moulard	5 days	5000	50,100,150,300
H2	2022	Qalyubia	Muscovy	7 days	9700	160, 250,350
H3	2022	Qalyubia	Muscovy	4 days	4000	120, 150,300
H4	2022	Sharqia	Pekin	12 days	5000	200,250,300
H5	2022	Qalyubia	moulard	10 days	3000	122,140,200
H6	2023	Qalyubia	Pekin	15 days	4000	10,20,50
H7	2023	Assuit	Muscovy	7 days	8000	80,160,280
H8	2023	Qalyubia	Pekin	10 days	1000	2,3,10

### Molecular analysis of infected duckling’s liver for the presence of DVH type I (DHAV-1 and DHAV-3) family *Picornaviridae*

Un-pooled liver samples from freshly dead suspected ducklings were molecularly tested by reverse transcription polymerase chain reaction (RT-PCR) using specific primers targeting DHAV-1 and DHAV-3 in family *Picornaviridae* that were suspected to be the cause of the hepatic lesion and the nervous manifestations^24^. Viral RNA was extracted, and the RT-PCR was done following the instructions. The positive controls used for DHV-1, DHV-3 kindly provided by Central laboratory for Evaluation of Veterinary Biologics.

### Molecular analysis of infected duckling’s liver for the presence of astrovirus infection

Forty liver samples from freshly dead suspected ducklings (5 liver from each farm, Table 1) were tested molecularly for the presence of astrovirus. As followed, RNA was extracted using 300 µl of liver homogenate using Trizol reagent (Thermo Scientific cat # 15596-026) following the manufacturing instructions. The purified RNA preparation was treated with DNase I (ThermoFisher cat #18068015) and stored at −80 °C till usage.

### Preparation of cDNA and PCR amplification

The first strand cDNA was synthesized from the purified, DNase-treated RNA samples using M-MuLV First Strand cDNA Synthesis (Biomatik cat# K5147) following the manufacture instructions. PCR amplification was performed using the high-fidelity polymerase (Phusion plus DNA polymerase, Thermos Fisher cat # F630XL) according to the manufacture instructions. Briefly, 5µl of the cDNA was mixed with 10µl of 5x fusion plus buffer, 200pM of each forward and reverse primers, 1µl of dNTPs (200mM each), 10µl of 5x fusion GC enhancer buffer and 0.5µl of fusion plus polymerase in 50µl reaction buffer. The reaction was denatured at 98°C/1 min then amplification was done for 35 cycles of denature at 98°C/10sec, annealing at 45°C/10sec and extension at 72°C/30 sec with final extension at 72°C/10 min. Forward primer 5’- GAY-TGG-ACH-MGH-TAY-GAY-GGH-ACH-ATH-CC-3’ and reverse primer 5’-YTT-HAC-CCACAT-HCC-RAA-3 used in the current study to amplify a fragment of approximately 434 nt encoding non-structural proteins and is highly conserved, which corresponds to nucleotides 3799 to 4233 in the genome of G4260 Avastrovirus 2 (accession number AB033998)^11^.

### Sequencing and Data analysis

The PCR product of Al-Sharqia isolated DAstV strain was electrophoresed on a 1% low melting agarose, stained with ethidium bromide and visualized under the UV transilluminator. A 100 bp DNA ladder (GenRuler DNA ladder enzynomics cat #DM001) was used to determine the size of the amplicons. The amplified specific band was sliced off and purified using gel purification kit (Biobasic-Canada cat #.BSC02S1) according to the manufacturer’s instructions. Sequencing reactions were performed in a M.J. Research PTC-225 Peltier Thermal Cycler using ABI PRISM 3730XL Analyzer Big DyeTM Terminator Cycle Sequencing Kits with AmpliTaq DNA polymerase (F.S. enzyme Applied Biosystems). Nucleotide sequence analyses were conducted with the LaserGene sequence analysis software package (LaserGene, version 15; DNAStar, Inc.). Alignments of the sequences were performed using the Clustal W module. Phylogenetic analysis was created using Maximum-likelihood (ML) using BLAST search module retrieved from GenBank records. The gene sequence was assigned as DAstV- VSVRI-CLEVB-2022 and submitted to the GenBank at the accession number PP105462.

### Virus isolation and adaptation on CAM of SPF- ECE

SPF- ECE were obtained from the national SPF poultry project at Kom Oshim, Fayoum, Egypt. The liver tissue of freshly dead (naturally infected) ducks was homogenized mechanically by PRO 200 homogenizer (Pro Scientific USA), the inoculum was tested for its sterility and inoculated according to OIE^24,30^. Four serial egg passages (using the liver homogenate of dead embryos) were done during virus adaptation on ECE. CAM of all egg passages were identified molecularly by RT-PCR as mentioned before identifying the presence of astrovirus.

### Sample preparation for virus detection by transmission electron microscopy (TEM)

Electron microscopy (EM) was used to observe the virus depending on previous studies^31^. For this, purified high titer virus was prepared according to Soliman et al.^32^. CAM homogenate from the fourth passage on ECE was first centrifuged at 14.000 rpm/10min/4°C to remove cell debris and high molecular weight proteins, then the supernatant virus was set onto sucrose cushion (3 mL 20% and 3 mL 50% sucrose prepared in TNE buffer [20 mM Tris-HCl (pH 7.6), 100 mM NaCl, 2 mM EDTA].) and centrifuged for 2 h at 30,000 rpm in Sorvall^®^ Surespin™ 630 swinging bucket ultracentrifuge rotor using Sorvall WX 100 ultracentrifuge (Thermo Fisher scientific, USA). The layer containing the virus was aspirated and the virus particles were sedimentated at 65,000 rpm/3 h/4°C. The virus sediment was re-suspended by gentle agitation in 1 mL of TNE (prepared with nuclease free water) overnight at 4 °C and stored at −80 °C. 100 µl of the purified sample was used for measuring the virus titer as mentioned before. The morphology of the virus was visualized using Field Transmission Microscopy. The virus sample was highly diluted with de-ionized (1:200 v/v) and mounted on a copper carbon-coated grid of 200 mesh, then stained with Phosphotungstic acid (PTA 2%) for 30 s and examined with a JEOL 2100 transmission electron microscope.

### Titration of the isolated astrovirus strain used for pathogenicity testing

To measure the virus titer, livers and CAM of the fourth passage on SPF -ECE were individually homogenized mechanically by PRO 200 homogenizer (Pro Scientific USA), diluted with buffered saline, and frozen and thawed three successive times, followed by centrifugation at 8000 rpm for 5 min, then the supernatant was collected.

According to Anon^33^, ten-fold serial dilutions from 10^− 1^ to 10^− 11^ of the collected supernatants of liver and CAM homogenates were prepared, and 0.1 ml of each dilution was inoculated at five SPF-ECE 11- days old via the CAM route. Eggs were kept for 7 days at 37 °C, then chilled to collect the CAM and record the lesion^34^. Egg infective dose fifty (EID50) of the virus was calculated according to Reed & Muench^35^.

### Pathogenicity testing in one day old ducklings

Due to the unavailability of SPF ducks in Egypt, twenty-one-day-old ducklings ‘star breed’ were obtained from the parent farm (Al-Wafaa Farm, Giza, Egypt). Ducklings were tested serologically to ensure the absence of any infection and other waterfowl-related viruses, especially avian influenza H5N1 subtype and DVH-1 following OIE^24,30^. Ducklings have been divided into two groups of 10 birds each. The first group (positive control) was inoculated via the oculo-nasal route with 0.5 ml (10^5^ EID50/µl) of the isolated virus while the second group (negative control) was inoculated with 0.5 ml of sterile phosphate buffered saline (pH 7.4) via the same route. Birds were kept in separate pens, and all requirements including bedding, lighting, and humidity were adjusted accordingly and supplied with open-access autoclaved water and feed (starter mash type). The study designed to observe the duckling for 10 days observation period, but all experimentally infected ducklings had died within 2 days post the experimental infection due to DAstV and during this period duckling were kept under strict observation for monitoring and recording clinical signs and death patterns.

### Histopathological examination

In the current study, samples of experimentally infected ducklings from liver, pancreas, spleen, heart, kidney, intestine and brain were collected. Samples were preserved in 10% neutral buffered formalin then passed through ascending alcohol grades, cleared in xylene then embedded in melted paraffin wax and cut at 5 μm thickness for hematoxylin and eosin (H and E) staining^36^.

## Results

### Clinical and pathological investigations

**Fig. 1:**
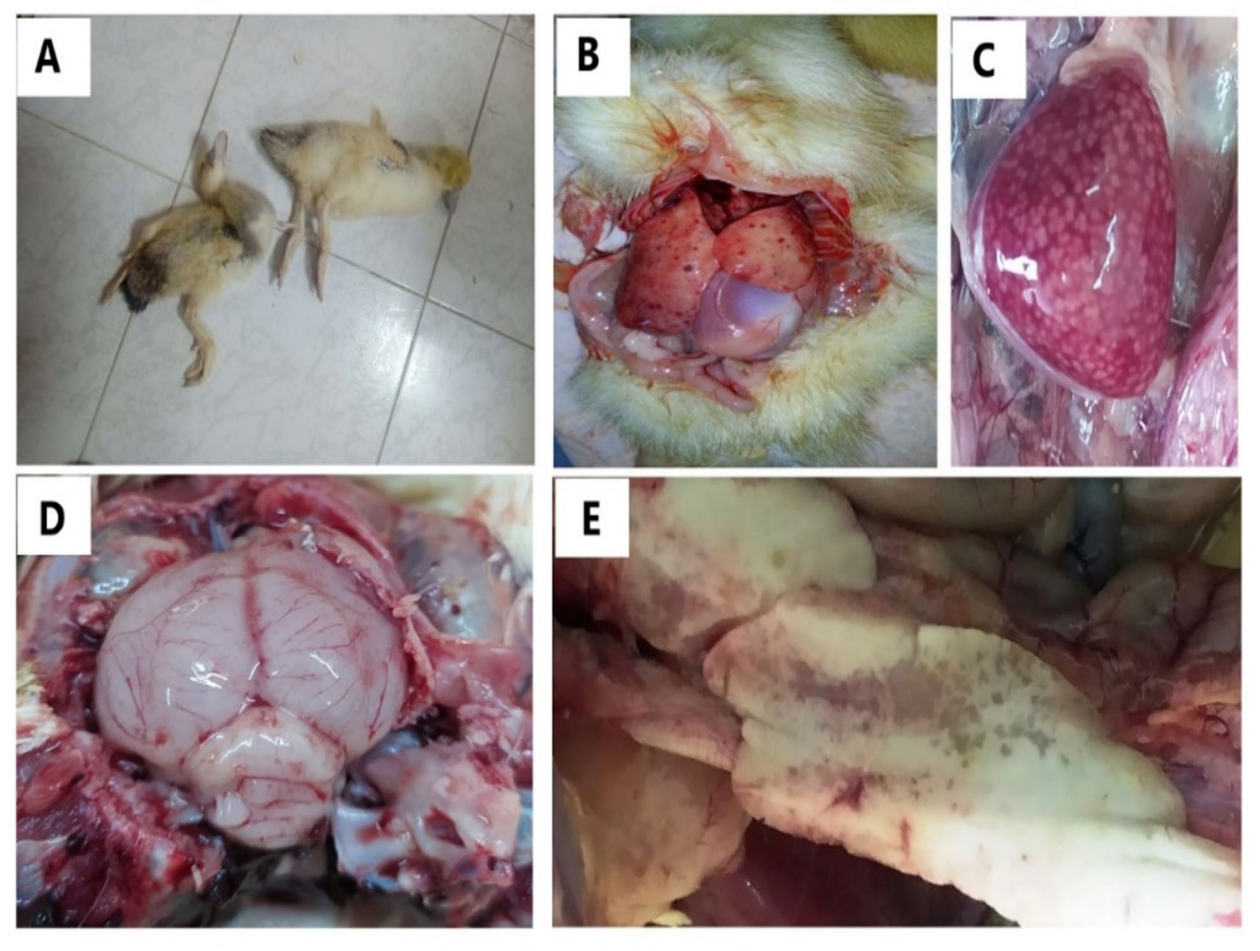
Clinical signs and PM lesions of field infection cases in ducklings; A: Dead birds showing nervous signs, B: Liver showing hemorrhage, C: Enlarged mottled spleen, D: Hemorrhage on brain meninges, & E: Pancreatic necrosis.

In the field cases, the infected ducklings showed typical clinical signs of DHV, lethargy, tremors, torticollis, opisthotonos, spasmodic paddling, laying on their back with sudden death. At necropsy, the collected samples showed hemorrhages in the liver, enlarged mottled spleen, hemorrhage on brain and necrosis in the pancreas (Fig. 1).

Under the experimental infection condition, the challenged birds showed a short course with signs of lethargy, tremors, neck deviation, lay on back, leg paddling followed by sudden deaths.

### Molecular detection by PCR

Due to the presence of nervous signs on the dead ducklings, there was a demand to test for the presence of H5N1 infection, and when tested by RT-PCR, the result was negative. The same negative result was obtained when liver samples were tested for the presence of DVH type I (DHAV-1 and DHAV-3) family *Picornaviridae*. The virus isolate identified as astrovirus (DAstV) when tested molecularly.

All liver samples collected from the three governorates (Al-Qalyubia, Al-Sharqia, and Assuit) were molecularly positive PCR targeting DAstV.

CAM of the third and fourth egg passage and liver of experimentally infected ducks showed positive 430 bp PCR amplicon size while the harvested greenish allantoic fluid of all passages was molecularly negative.

### Sequence and phylogenetic analysis

**Fig. 2:**
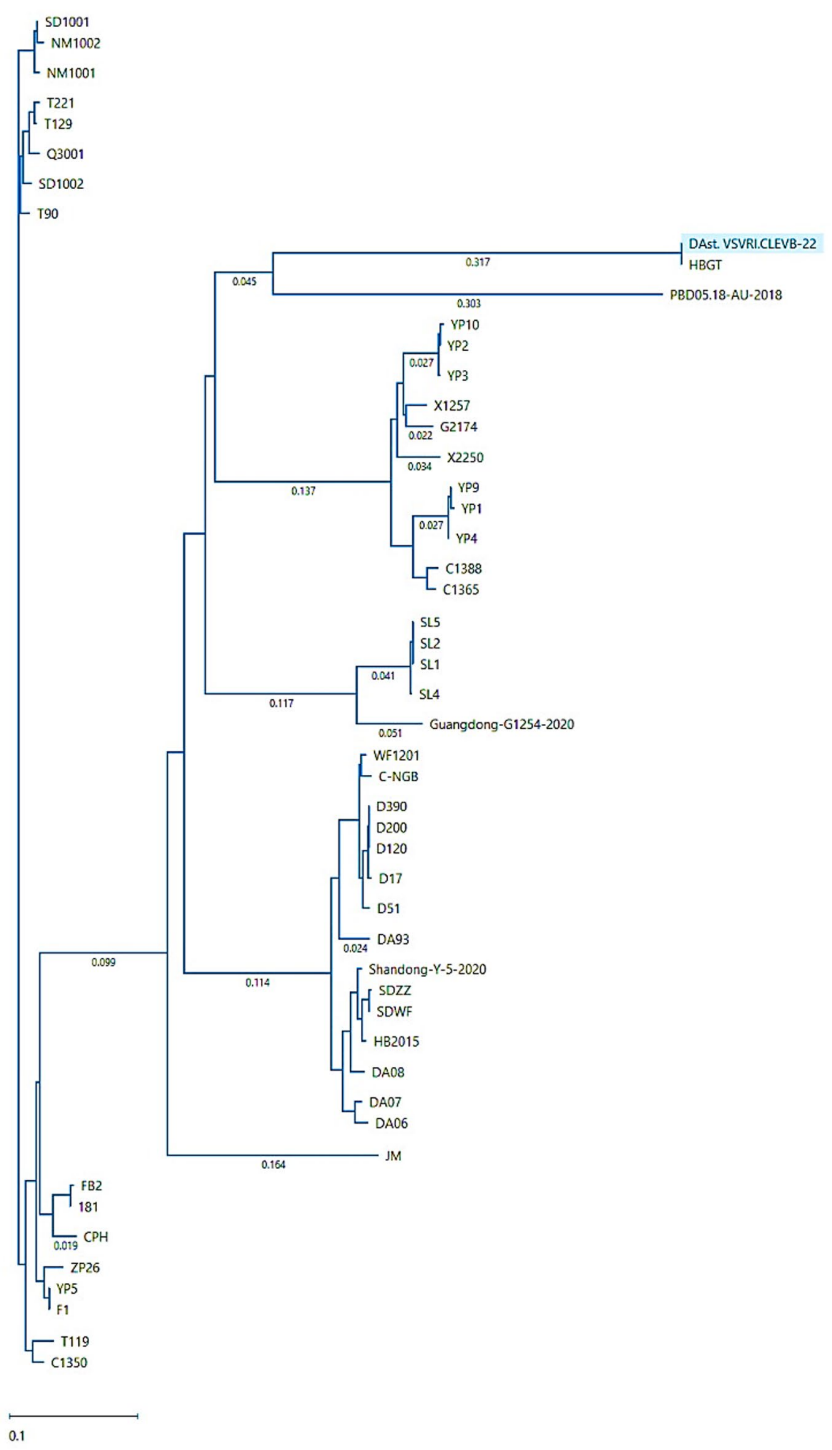
Phylogenetic tree of the isolated DAstV ORF1b encoding non-structural proteins (VSVRI-CLEVB-2022) showed that, the isolated virus is clustered with HBGT-China-2014 and PD05.18-AU2018 and shared high nucleotide homology (100% and 99.4%) with both strains respectively.

The nucleotide sequence targeting ORF 1b of the isolated DAstV from Al-Sharqia governorate was published in GenBank with accession number PP105462. The phylogenetic trees of the amino acid showed that the isolated DAstV virus was clustered with the HBGT- China-2014 strain KX290465.1 (Fig. 2) and showed a 100% similarity when observed in the similarity matrix.

### Virus isolation on CAM of SPF-ECE

The tested inoculum was sterile and the CAM of inoculated SPF-ECE showed pinpoint necrosis accompanied by thickness observed at the first and second passages, crusty appearance of the CAM observed by the third and the fourth passage. The embryos were hemorrhagic and showed evidence of ascites; the liver of the embryos, allantoic fluid, and yolk showed a greenish color. At the fourth passage, CAM and liver lesions were recorded on day 5 post-inoculation, and embryos deaths were recorded on the 8th day post-inoculation and continued to the 10th day. It was so clear that the severity of the lesion, including the degree of the greenish colour of the liver and allantoic fluid, as well as the liver lesions, was exaggerated by the fourth passage.

### Titration of Virus strain used for pathogenicity testing

**Fig. 3:**
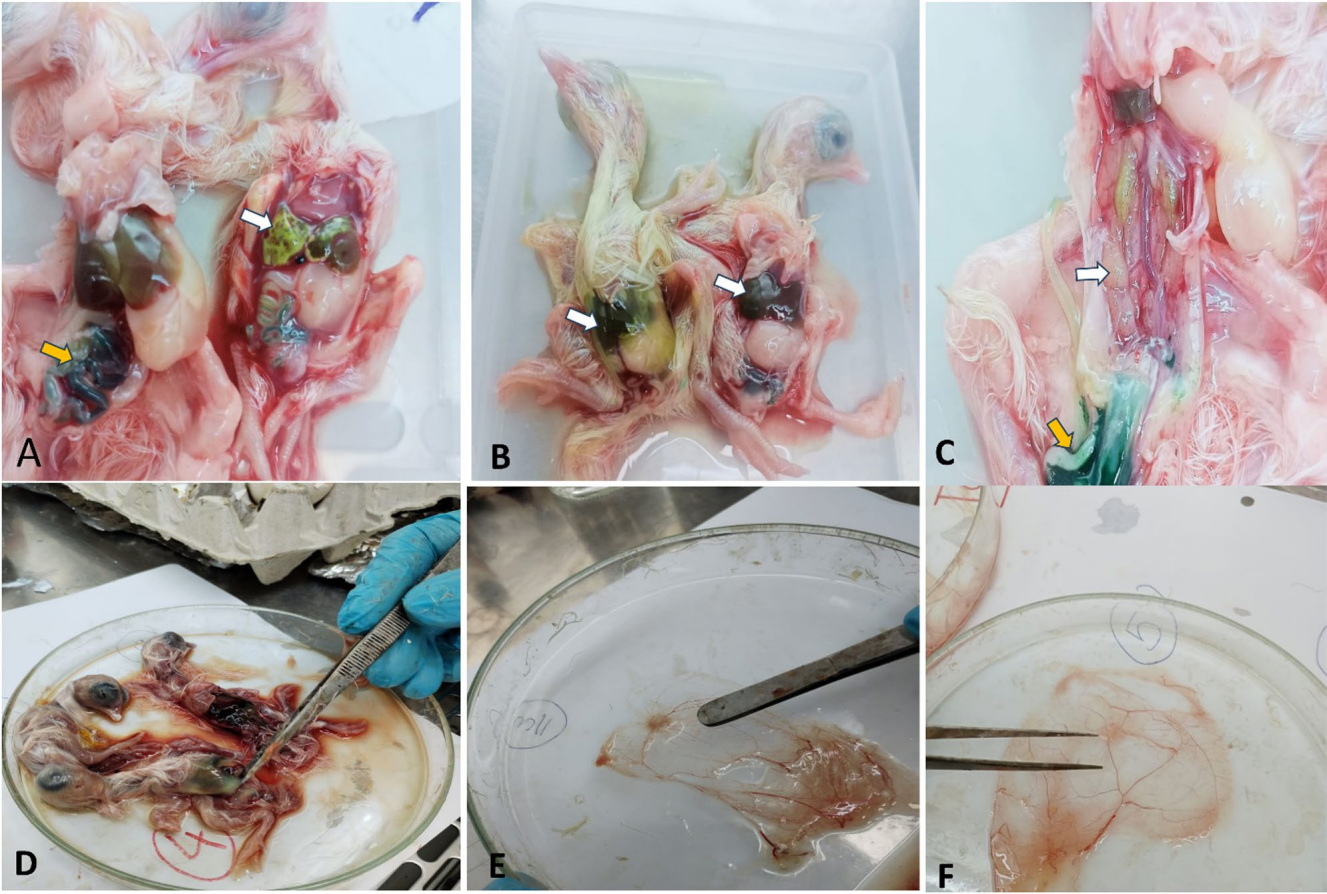
Titration of DAstV on ECE: A & B: Lesion due to 10^− 1^ virus dose showing necrosis with discoloration of the liver (white arrow), greenish discoloration in the intestine (yellow arrow); C: Lesion due to 10^− 2^ virus dose showing paleness with nephrosis in the chicken embryo kidney (white arrow), greenish discoloration in the intestine (yellow arrow); D: Lesions due to 10^− 3^ virus dose showing necrosis & greenish discoloration of liver; E & F: Showing necrotic lesion on CAM due to 10^− 5^ virus dose.

In all inoculated eggs (5 eggs/dilution), chorio-allantoic membrane of dilutions (10^− 1^:10^− 8^) showed hepatic necrotic lesions; the liver of embryos showed different discoloration varying according to the inoculated dilution; the kidneys as well showed nephritis and nephrosis; and allantoic fluid showed variable degrees of greenish colour, also the CAM showed necrotic lesion due to 10^− 5^ virus dose (Fig. 3). The virus titer was 10^8^/µl and 10^11^/µl for liver and CAM isolates respectively.

### Pathogenicity testing in day old ducklings

#### Clinical signs, pattern of the deaths of ducklings and gross lesions post infection

All the tested ducklings were serologically negative for avian influenza H5N1 subtype and DVH-1.

After 25 h PI, ducklings began to exhibit loss of balance and incoordination in movement; six hours later (31 h PI), three ducklings died after showing the typical indications of viral hepatitis (paddling spasmodically with a stretched neck); 38 h PI, another three ducklings died with the same previous pattern; and 50 h PI, the remaining four ducklings died. Shortly before their deaths, all ducklings developed greenish, watery diarrhoea. The gross lesion of ducklings showed paintbrush hemorrhagic bands on the liver surface, clear haemorrhage on the meninges, splenomegaly, nephritis and nephrosis, enteritis, and an unabsorbed yolk sac.

#### TEM

**Fig. 4:**
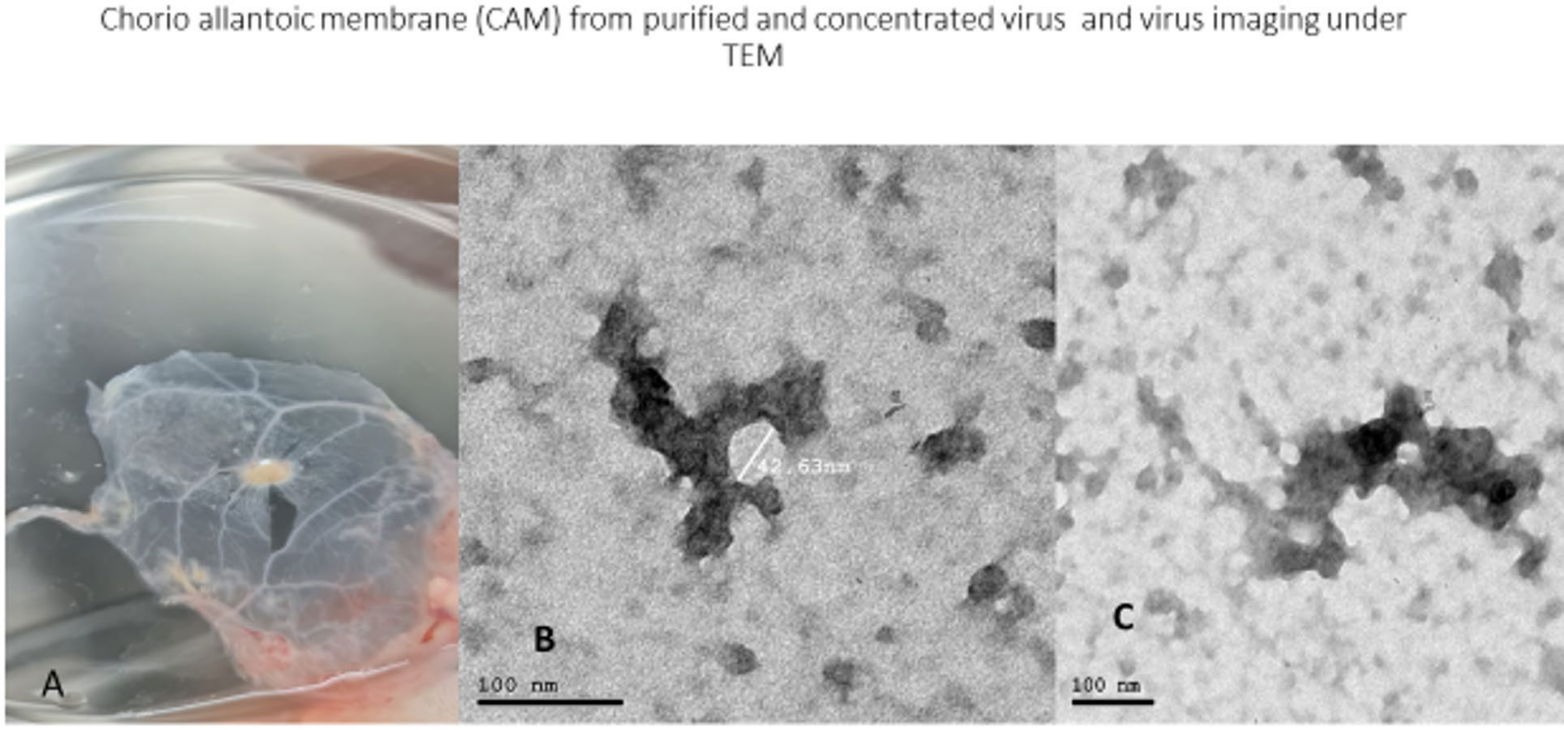
Duck astrovirus particles inoculated in ECE show A: clear necrosis on CAM. B, C: purified and concentrated virus particles imaging under TEM.

EM examination of the purified high titer virus (10^11^/µl) showed clear six-pointed end virus shown with 42 nm size which is characteristic to astrovirus (Fig. 4).

#### Histopathological findings

**Fig. 5:**
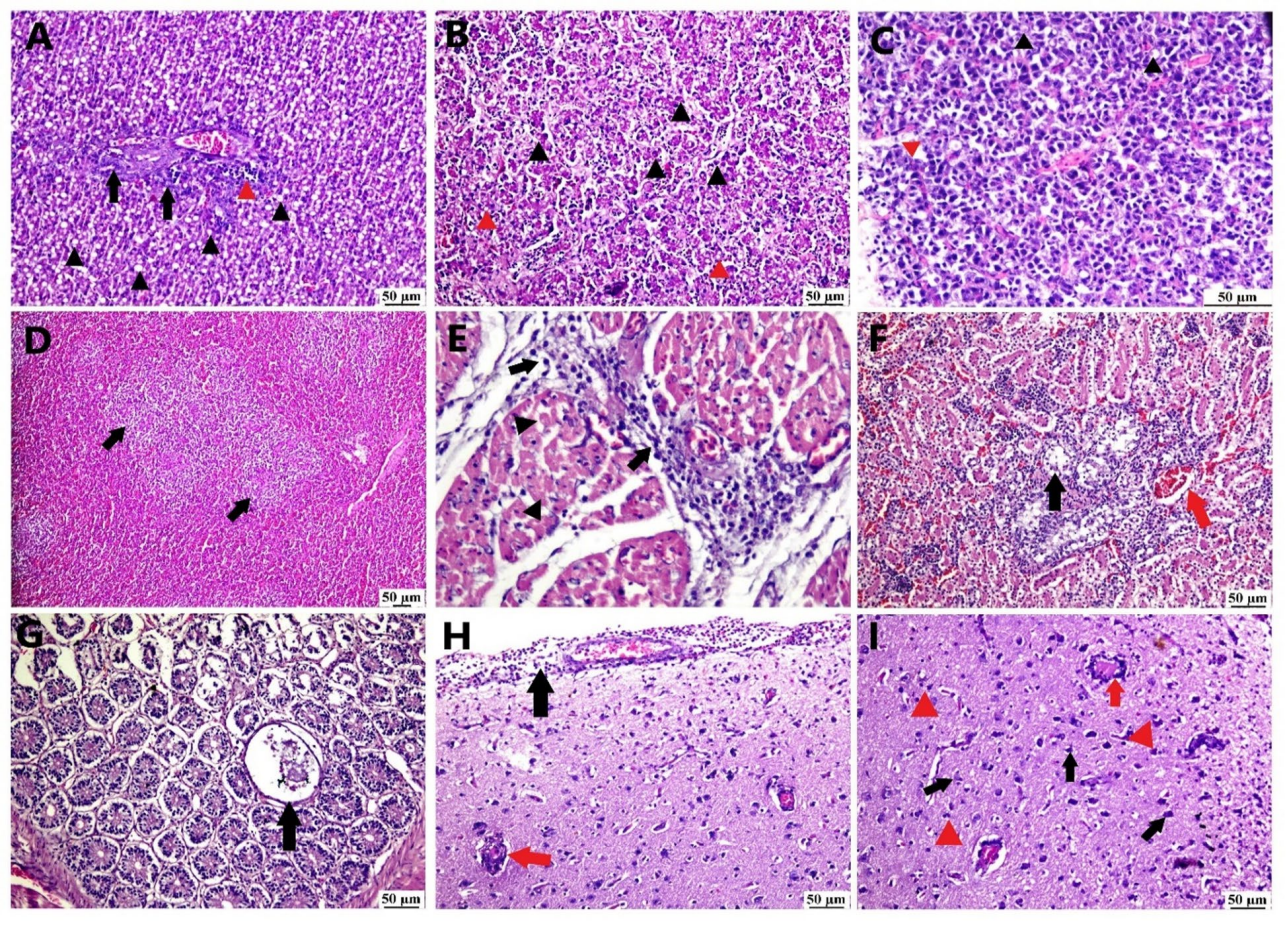
Demonstrating histopathological variations associated with experimentally infected ducklings with DAstV (H&E): (A) Liver showing micro and macrovesicular steatosis (black arrowhead) with focal aggregation of inflammatory cells (red arrowhead) and bile ductular hyperplasia (black arrow), (B) Hepatocellular apoptosis (black arrowhead), hemorrhage and microvesicular steatosis (red arrowhead), (C) Pancreas, acinar cells are shrunken that devoid of zymogen granules with apoptotic vacuoles (black arrowhead) and necrosis (red arrowhead) of acinar cells, (D) lymphoid depletion of splenic lymphoid follicles (black arrow) (E) heart showing lymphocytic infiltration (black arrow) around blood vessels and between necrosed myocytes (black arrowhead), (F) Kidney, necrosis and degeneration of tubular lining epithelium (black arrow) beside congestion of peritubular blood vessels (red arrow), (G) Intestine, cryptic cystic dilatation (black arrow) that lined with attenuated epithelium and contain intraluminal cellular debris, (H) Brain, thickening of meninges with lymphocytic infiltrations (black arrow) with thickening of blood vessels (red arrow), (I) Brain, neuronal degeneration (black arrow) with gliosis (red arrowhead), vasculitis and perivascular lymphocytic cuffing (red arrow).

Concerning histopathological variations of pathogenicity study, different tissues have been assessed in Fig. 5. Hepatic sections showed discontinuity of hepatic cords with marked hyperemia of blood vessels and sinusoids associated with hemorrhage. Hepatocellular necrosis and apoptosis were detected with micro-vesicular or macro-vesicular steatosis accompanied by intense lympho-histiocytic cells infiltrated between necrosed hepatocytes and periportal. Hyperplasia of the biliary epithelium was observed. Regarding the examined pancreatic tissue, depletion of zymogen granules was detected besides congestion with focal pancreatic apoptosis, necrosis and inflammation. Examined sections of the spleen exhibited congestion of the red pulp and depletion of the white pulp with lymphocytolysis. Degeneration and necrosis of renal tubules were present with subcapsular hemorrhage, congestion of interstitial blood vessels and heterophilic infiltration.

Epicardial hemorrhage and focal myocardial necrosis with lymphocytic infiltration were observed in addition to congestion of blood vessels. While intestinal sections revealed mucosal necrosis with mononuclear cells infiltration in lamina propria and submucosa, cystic dilatation of the crypt was also seen. Brain sections displayed meningitis, additionally, neuronal degeneration with gliosis, thickening of blood vessels and perivascular lymphocytic cuffing were obvious. No microscopical lesions were present in the control negative group.

## Discussion

Viral hepatitis is a complex disease syndrome that affects the liver of poultry^4^. It is caused by many viruses belonging to different families, including DHAV, DAstV-I, DAstV-II, fowl adenoviruses (FAdV), and turkey hepatitis virus (THV)^4^. Although various hepatitis viruses target the same organ, the liver, they each have distinct clinical and molecular characteristics^14^, except for DAstV, which causes acute and fatal infections in ducklings with a rapid decline in 1–2 h with clinical and pathologic symptoms that are nearly identical to DHAV^29^. Since the late 1960 s where Refaie et al.^37^. recorded the first DHAV infection in Egypt, several studies and research had been conducted to discuss the epidemiological and the molecular situation of DHAV in Egypt^24,38^. Egyptian DHAV-1 isolates found to be clustered in genetic group 4 with virulent Chinese strains^39^, after that Mansour et al.^40^. classify the Egyptian DHAV-1 isolates into three subgroups (A, B, and C) under genetic group 4 based on their geographical distribution.

Astrovirus infections have a great impact on poultry production due to their typically fatal effect on young birds and their ability to transmit vertically^20^. That is why they are usually associated with the hatchery disease of broiler chicks, runted chicks, and dwarfed pale chicks^41^. The clinical signs associated with the astrovirus infection differ according to the species, where in chickens it is known as malabsorption syndrome, gosling gout diseases and fatal hepatitis in young duckling^42^.

Regarding astrovirus infection in Egypt the reports are limited, Sallam et al.^43^. found that chicken astrovirus (CAstV) Egyptian isolates shared the highest nucleotide homology (93%) with the Korean isolate Kr/ADL102655-1/2010 and showed the most distant relation to the Indian isolate Indovax/APF/1319 with 82–83% homology. Moreover, El Taweel et al.^44^. recorded astroviruses as common in bats, wild birds, and humans in Egypt. Until now, no clear data was available on the genetic diversity of circulating astroviruses in Egypt.

Previous literatures emphasized the endemicity of DHAV-3 belongs to family *Picornaviridae* as a main cause of DVH in Egypt^28^. During sample collection in this study, ducklings were suffering from nervous manifestations and severe hepatic lesions during postmortem examinations and for this reason the duck’s livers were tested for DHAV-3 to be the cause of those clinical infections.

In the current study, when samples tested molecularly targeting the *Picornaviridae* family, the results were negative in all examined livers, also the isolation of the virus on allantoic sac of SPF ECE showed no lesions, and so liver samples were then tested molecularly for astrovirus infection and the molecular identification of the astrovirus showed positive PCR amplification of 434 bp, which corresponds to nucleotides 3799 to 4233 in the genome of G4260 Avastrovirus 2. After that, astrovirus was isolated on CAM according to OIE recommendation^24^ and Xu et al.^45^. results of isolation showed a clear necrotic crusty CAM, and the presence of the DAstV was positive when tested molecularly by PCR test, on the other hand, the allantoic fluid showed green discoloration that was molecularly negative DAstV when tested by PCR, this green discoloration may be explained due to the pathogenesis of the virus which cause extensive hepatic necrosis, cholangitis and proliferation of the bile duct epithelium and; the extent of bile duct hyperplasia may be somewhat greater than with DHAV^24^. These results agree with the study of Liu et al.^46^. they recorded formation of severe lesions on CAM of duck eggs, these results together with our obtained results reflect that the infection of CAM and the embryos may be a great cause for vertical transmission of astrovirus in hatchery. Another route for inoculation was recorded by Sallam et al.^43^. where isolation of chicken astrovirus was performed through intra yolk inoculation of 5–7 days old ECE, their results showed severe embryonic hemorrhages, edema, and dwarfism.

Although TEM has been replaced by many other sensitive diagnostic methods due to its nanometer scale resolution, it is still considered the only imaging technology that allows direct visualization of viruses especially during the preliminary identification of unknown viral agents^47^. Hoshino et al.^48^ detected astroviruses in feces of a cat suffering from diarrhea and the viral particles showed roughly spherical appearance in outline, its size ranged from 27.5 nm to 29.4 nm in diameter and the surface structure showed characteristic appearance of five- or six-pointed stars and this star-shaped structure was not seen on all particles.

Sensitivity of EM is also dependent on presence of high virus concentration in the examined sample^49^ that is why the authors use this high virus concentration (10^11^/µl EID50). TEM showed the identified virus was up to 41 nm in diameter, with a distinctive five- or six-pointed star on its surface with well-defined surface spikes.

The same result was clear during the study of Dryden et al.^50^ where the structure of mature and immature astrovirus of human was studied and found the size of the virus showed solid capsid shell ~ 35 nm in diameter (~ 44 nm with spikes), also ICTV^13^ stated that, virions shed in feces are 28–30 nm in diameter, spherical in shape and non-enveloped.

The astrovirus infections that affect chickens and other animal species, including humans, share many characteristics^42^. During this study the experimentally challenged duckling with astrovirus showed clear nervous signs including loss of balance, spasmodically paddling with a stretched neck and shortly before their deaths, all ducklings developed watery greenish diarrhoea. Frémond et al.^51^**&** Sato et al.^52^ emphasis the progression of astrovirus-associated encephalitis. Also, Raji & Omar^42^ in a parallel study in chickens, found that chicken astrovirus can induce runting-stunting syndrome, diarrhea, renal pathology associated with gout, and it also cause white chick syndrome in broiler chicks. In the current research there were numerous histopathological changes that have been recorded in both field and experimental cases due to duck astrovirus in ducklings’ liver, spleen, pancreas, kidney, intestine and brain. Similar histopathological changes have been reported by Sallam et al.^43^. including enteritis, decrease of villi length, proventriculitis, hepatic necrosis, myocarditis, pericarditis, and nephritis with deposition of urate in chickens infected with chicken astrovirus.

Sequence analysis of the ORF1b of the isolated DAstV shared the highest nucleotide homology (100% and 99.4%) with HBGT-China-2014 KX290465.1and PD05.18-AU2018 #MT894395 respectively. In a parallel study in chickens, Elena et al.^53^ mentioned the importance of full genome sequence of astrovirus to follow the genetic diversity of the virus among different avian species.

Finally, the transmission of astrovirus among different avian species may contribute to the environmental contamination that occurs primarily through contaminated water, food, soil, and surfaces^54^. Infected birds shed viruses through feces, respiratory secretions, and bodily fluids, contaminating shared environments such as wetlands, farms, and feeding areas^54^. Water sources play a crucial role in spreading avian viruses, as many bird species congregate in the same habitats^54^. Also, mixed rearing systems and the small distances between different farms increase the incidence of diseases transmission among different avian species. Additionally, fomites such as feathers, nesting materials, and human activities can facilitate cross-species transmission^54^. Environmental persistence of astrovirus further enhances their spread, increasing the risk of outbreaks among wild and domestic bird populations^55^. In addition, the genetic structure and diversity of astrovirus enable the interspecies transmissions of the virus and enable the virus to infect a variety of species as human, duck, chicken, turkey, pigs, ovine, bovines, dog, red deer, mink, dolphin etc^56^,. The previously mentioned data declare that astrovirus was transmitted in Egypt among different species including human, bats, chickens, wild birds etc^44,57^., &^43^ and recently ducks as recorded in the current study.

**Conclusion**.

In conclusion, we isolate and identify DAstV to be one of the causative agents of acute duck hepatitis in Egypt for the first time, and the virus designated as DAstV- VSVRI-CLEVB-2022. Based on molecular detection, phylogenetic analysis, electron microscopy and pathogenicity testing. More investigations are needed to study the genetic diversity of circulating Duck astrovirus in Egypt, also it is recommended to prepare local autogenous vaccine from the isolated strain as a preventive strategy side by side with the biosecurity measures to control such a problem and evaluate its efficacy against field challenge to overcome the mortalities induced by astrovirus in ducklings.

M.A.N.G.; E.M.S.E.: Virus isolation and Molecular studies; M.A.E and M.R.M.: Histopathological studies; H.M.S. and K.S.: samples collection; H.M.S., E.M.S.E, MA.N.G.: design the experiment; all authors share writing and revision of the manuscript.

## Data Availability

The nucleotide sequence targeting ORF 1b of the isolated astrovirus was assigned as DAstV- VSVRI-CLEVB-2022 and submitted to GenBank at the accession number PP105462 and other resulting data during this study is included in this manuscript. Any explanation of additional datasets in the current study is available from the corresponding author upon reasonable request.

## Declarations

### Approval of animal experiments

All experimental protocols were approved and assessed by the Animal Care and Use Committee of Cairo University’s Faculty of Veterinary Medicine and approved by the Institutional Animal Care and Use Committee (IACUC) with code ‘03162023704’.
